# 1′-(4-Bromo­phen­yl)-4′-{4-[(2-oxo-1,2,3,4-tetra­hydro­naphthalen-2-yl­idene)meth­yl]phen­yl}-3′′,4′′-dihydro­acenaphthylene-1-spiro-2′-pyrrolidine-3′-spiro-2′′-naphthalene-2,1′′(1*H*,2′′*H*)-dione

**DOI:** 10.1107/S1600536810040171

**Published:** 2010-10-13

**Authors:** B. Saravanan, R. Rajesh, R. Raghunathan, G. Chakkaravarthi, V. Manivannan

**Affiliations:** a Department of Physics, PRIST University, Thanjavur 614 904, Tamil Nadu, India; b Department of Organic Chemistry, University of Madras, Guindy Campus, Chennai 600 025, India; c Department of Physics, CPCL Polytechnic College, Chennai 600 068, India; d Department of Research and Development, PRIST University, Thanjavur 613 403, Tamil Nadu, India

## Abstract

In the title compound, C_47_H_34_BrNO_3_, the central benzene ring makes a dihedral angle of 42.71 (7)° with the bromo­phenyl ring. The pyrrolidine ring adopts an envelope conformation. The mol­ecular structure is stabilized by weak intra­molecular C—H⋯O inter­actions and the crystal packing is stabilized by weak inter­molecular C—H⋯π inter­actions.

## Related literature

For the biological activity of pyrrolidine derivatives, see: Amalraj *et al.* (2003 ▶); Daly *et al.* (1986 ▶). For related structures, see: Aravindan *et al.* (2004 ▶); Kumar *et al.* (2006 ▶). For graph-set notation, see: Bernstein *et al.* (1995 ▶).
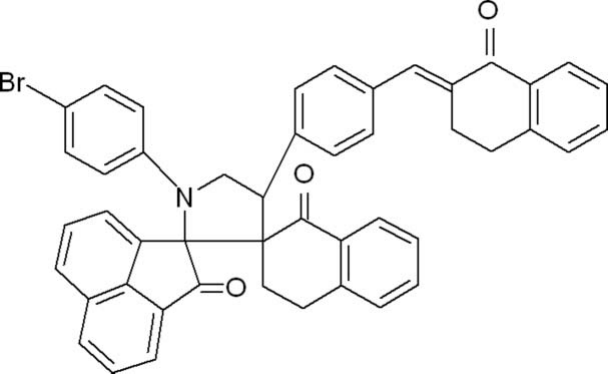


## Experimental

### 

#### Crystal data

C_47_H_34_BrNO_3_
*M* *_r_* = 740.38
Triclinic, 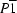

*a* = 8.4178 (2) Å
*b* = 13.2352 (3) Å
*c* = 15.9610 (3) Å
α = 98.143 (1)°
β = 92.744 (2)°
γ = 100.944 (1)°
*V* = 1723.17 (7) Å^3^
*Z* = 2
Mo *K*α radiation
μ = 1.24 mm^−1^
*T* = 295 K
0.20 × 0.19 × 0.18 mm

#### Data collection

Bruker Kappa APEXII diffractometer
Absorption correction: multi-scan (*SADABS*; Sheldrick, 1996 ▶) *T*_min_ = 0.719, *T*_max_ = 0.779
30711 measured reflections
6410 independent reflections
4539 reflections with *I* > 2σ(*I*)
*R*_int_ = 0.034

#### Refinement

*R*[*F* ^2^ > 2σ(*F* ^2^)] = 0.037
*wR*(*F* ^2^) = 0.098
*S* = 1.03
6410 reflections
469 parameters
H-atom parameters constrained
Δρ_max_ = 0.32 e Å^−3^
Δρ_min_ = −0.53 e Å^−3^

### 

Data collection: *APEX2* (Bruker, 2004 ▶); cell refinement: *SAINT* (Bruker, 2004 ▶); data reduction: *SAINT*; program(s) used to solve structure: *SHELXS97* (Sheldrick, 2008 ▶); program(s) used to refine structure: *SHELXL97* (Sheldrick, 2008 ▶); molecular graphics: *PLATON* (Spek, 2009 ▶); software used to prepare material for publication: *SHELXL97*.

## Supplementary Material

Crystal structure: contains datablocks global, I. DOI: 10.1107/S1600536810040171/is2611sup1.cif

Structure factors: contains datablocks I. DOI: 10.1107/S1600536810040171/is2611Isup2.hkl

Additional supplementary materials:  crystallographic information; 3D view; checkCIF report

## Figures and Tables

**Table 1:** Hydrogen-bond geometry (Å, °) *Cg*1 and *Cg*2 are the centroids of the C3–C8 and C12–C17 rings, respectively.

*D*—H⋯*A*	*D*—H	H⋯*A*	*D*⋯*A*	*D*—H⋯*A*
C22—H22*B*⋯O3	0.97	2.55	3.026 (3)	111
C23—H23*B*⋯O3	0.97	2.40	3.113 (3)	130
C17—H17⋯*Cg*1^i^	0.93	2.98	3.720 (3)	137
C26—H26⋯*Cg*2^ii^	0.93	3.00	3.925 (3)	178

Symmetry codes: (i) 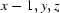; (ii) 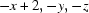.

**Fig. 1.**
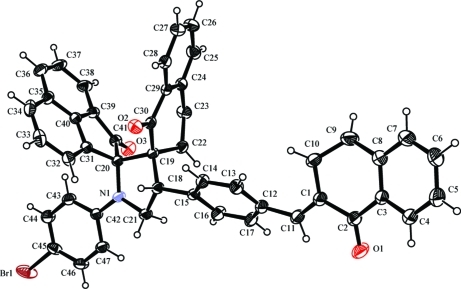
The molecular structure of (I), with atom labels and 30% probability displacement ellipsoids for non-H atoms.

## supplementary crystallographic information

### Comment

Highly substituted pyrrolidines have gained much prominence since they form the central skeleton of many natural products (Daly *et al.*, 1986). Substituted pyrrolidine compounds possess antimicrobial and antifungal activities against various pathogens (Amalraj *et al.*, 2003).

The geometric parameters of the title compound (Fig. 1) agree well with reported similar structures (Aravindan *et al.*, 2004; Kumar *et al.*, 2006). The benzene ring (C12–C17) makes a dihedral angle of 42.71 (7)° with the bromophenyl ring (C42–C46). The sum of bond angles around N1 [359.9 (2)°] indicates that N1 is *sp*^2^-hybridized.

The molecular structure is stabilized by weak intramolecular C—H···O interactions and the crystal packing is stabilized by weak C—H···π [C17—H17···*Cg*1 (-1 + *x*, *y*, *z*) distance of 3.720 (3)Å and C26—H26···*Cg*2 (2 - *x*, -*y*, -*z*) distance of 3.925 (3)Å (*Cg*1 and *Cg*2 are the centroid of the rings defined by the atoms C3–C8 and C12–C17, respectively) interactions. The intramolecular interactions C22—H22B···O3 and C23—H23B···O3 generate S(6) and S(7) graph set motifs, respectively (Bernstein *et al.*, 1995).

### Experimental

To the solution of acenaphthequinone (3) (1.1 mmol), *N*-(*p*-bromo) phenyl glycine (2) (1.1 mmol) and 1,4-bis(3',4'-dihydro-1'- oxonaphthalen-2'-ylidene)benzene (1) (1.0 mmol) was refluxed in dry toluene. Completion of the reaction was evidenced by TLC analysis. The solvent was then removed in vacuum, diluted in dichloromethane, washed with water, and brine. The organic layer was separated and dried over Na_2_SO_4_.The organic solvent was removed and the residue was subjected to column chromatography using hexane/ethyl acetate (6:4) as eluent afforded the cycloadduct.

### Refinement

H atoms were positioned geometrically and refined using riding model with C—H = 0.93 Å and *U*_iso_(H) = 1.2*U*_eq_(C) for aromatic C—H, C—H = 0.98 Å and *U*_iso_(H) = 1.2*U*_eq_(C) for C—H, C—H = 0.97 Å and *U*_iso_(H) = 1.2*U*_eq_(C) for CH_2_.

### Crystal data

**Table:**

C_47_H_34_BrNO_3_	*Z* = 2
*M**_r_* = 740.38	*F*(000) = 764
Triclinic, *P*1	*D*_x_ = 1.427 Mg m^−^^3^
Hall symbol: -P 1	Mo *K*α radiation, λ = 0.71073 Å
*a* = 8.4178 (2) Å	Cell parameters from 8389 reflections
*b* = 13.2352 (3) Å	θ = 2.2–25.3°
*c* = 15.9610 (3) Å	µ = 1.24 mm^−^^1^
α = 98.143 (1)°	*T* = 295 K
β = 92.744 (2)°	Block, colourless
γ = 100.944 (1)°	0.20 × 0.19 × 0.18 mm
*V* = 1723.17 (7) Å^3^	

### Data collection

**Table:**

Bruker Kappa APEXII diffractometer	6410 independent reflections
Radiation source: fine-focus sealed tube	4539 reflections with *I* > 2σ(*I*)
graphite	*R*_int_ = 0.034
ω and φ scan	θ_max_ = 25.5°, θ_min_ = 2.2°
Absorption correction: multi-scan (*SADABS*; Sheldrick, 1996)	*h* = −10→10
*T*_min_ = 0.719, *T*_max_ = 0.779	*k* = −16→15
30711 measured reflections	*l* = −19→19

### Refinement

**Table:**

Refinement on *F*^2^	Primary atom site location: structure-invariant direct methods
Least-squares matrix: full	Secondary atom site location: difference Fourier map
*R*[*F*^2^ > 2σ(*F*^2^)] = 0.037	Hydrogen site location: inferred from neighbouring sites
*wR*(*F*^2^) = 0.098	H-atom parameters constrained
*S* = 1.03	*w* = 1/[σ^2^(*F*_o_^2^) + (0.0431*P*)^2^ + 0.5268*P*] where *P* = (*F*_o_^2^ + 2*F*_c_^2^)/3
6410 reflections	(Δ/σ)_max_ < 0.001
469 parameters	Δρ_max_ = 0.32 e Å^−^^3^
0 restraints	Δρ_min_ = −0.53 e Å^−^^3^

### Fractional atomic coordinates and isotropic or equivalent isotropic displacement parameters (Å^2^)

**Table:**

	*x*	*y*	*z*	*U*_iso_*/*U*_eq_	
Br1	0.15843 (4)	0.56227 (3)	0.415272 (18)	0.09413 (16)	
O1	1.4073 (2)	−0.15001 (17)	0.50404 (12)	0.0809 (6)	
O2	1.06900 (18)	0.31498 (12)	0.14876 (10)	0.0482 (4)	
O3	0.43369 (19)	0.21371 (13)	0.13308 (10)	0.0546 (4)	
N1	0.6591 (2)	0.32198 (14)	0.27716 (10)	0.0445 (5)	
C1	1.3657 (3)	−0.08403 (18)	0.37579 (14)	0.0497 (6)	
C2	1.4467 (3)	−0.14275 (19)	0.43260 (15)	0.0538 (6)	
C3	1.5830 (3)	−0.18953 (17)	0.39993 (14)	0.0485 (6)	
C4	1.6890 (3)	−0.2189 (2)	0.45546 (16)	0.0606 (7)	
H4	1.6733	−0.2103	0.5131	0.073*	
C5	1.8182 (4)	−0.2610 (2)	0.42732 (19)	0.0724 (8)	
H5	1.8905	−0.2793	0.4656	0.087*	
C6	1.8381 (4)	−0.2753 (2)	0.3422 (2)	0.0805 (9)	
H6	1.9238	−0.3047	0.3224	0.097*	
C7	1.7332 (4)	−0.2469 (2)	0.28582 (18)	0.0775 (9)	
H7	1.7483	−0.2577	0.2281	0.093*	
C8	1.6049 (3)	−0.2023 (2)	0.31344 (16)	0.0605 (7)	
C9	1.4889 (4)	−0.1689 (3)	0.25318 (17)	0.0819 (9)	
H9A	1.5410	−0.1566	0.2016	0.098*	
H9B	1.3942	−0.2243	0.2381	0.098*	
C10	1.4366 (4)	−0.0714 (2)	0.29222 (16)	0.0660 (7)	
H10A	1.3564	−0.0547	0.2536	0.079*	
H10B	1.5294	−0.0140	0.3009	0.079*	
C11	1.2418 (3)	−0.04406 (19)	0.40358 (15)	0.0526 (6)	
H11	1.2052	−0.0639	0.4540	0.063*	
C12	1.1534 (3)	0.02685 (17)	0.36698 (14)	0.0453 (5)	
C13	1.2262 (3)	0.10465 (17)	0.32291 (14)	0.0470 (6)	
H13	1.3342	0.1091	0.3111	0.056*	
C14	1.1411 (3)	0.17533 (17)	0.29635 (14)	0.0436 (5)	
H14	1.1933	0.2269	0.2675	0.052*	
C15	0.9800 (3)	0.17107 (16)	0.31169 (12)	0.0387 (5)	
C16	0.9073 (3)	0.09371 (19)	0.35579 (15)	0.0516 (6)	
H16	0.7991	0.0890	0.3673	0.062*	
C17	0.9932 (3)	0.02350 (19)	0.38283 (15)	0.0527 (6)	
H17	0.9415	−0.0273	0.4125	0.063*	
C18	0.8906 (3)	0.25063 (17)	0.28252 (12)	0.0383 (5)	
H18	0.9696	0.3164	0.2878	0.046*	
C19	0.8163 (2)	0.22580 (15)	0.18853 (12)	0.0334 (4)	
C20	0.7048 (2)	0.31159 (16)	0.19066 (12)	0.0355 (5)	
C21	0.7503 (3)	0.27266 (18)	0.33271 (13)	0.0455 (5)	
H21A	0.6849	0.2087	0.3453	0.055*	
H21B	0.7885	0.3188	0.3855	0.055*	
C22	0.7200 (3)	0.11361 (16)	0.16731 (13)	0.0419 (5)	
H22A	0.7831	0.0684	0.1905	0.050*	
H22B	0.6208	0.1091	0.1962	0.050*	
C23	0.6752 (3)	0.07172 (18)	0.07383 (14)	0.0490 (6)	
H23A	0.6373	−0.0031	0.0673	0.059*	
H23B	0.5863	0.1018	0.0543	0.059*	
C24	0.8125 (3)	0.09469 (17)	0.01929 (13)	0.0417 (5)	
C25	0.8106 (3)	0.03584 (19)	−0.06084 (15)	0.0557 (6)	
H25	0.7222	−0.0173	−0.0808	0.067*	
C26	0.9385 (4)	0.0560 (2)	−0.11032 (16)	0.0628 (7)	
H26	0.9348	0.0175	−0.1641	0.075*	
C27	1.0711 (3)	0.1320 (2)	−0.08132 (16)	0.0593 (7)	
H27	1.1581	0.1440	−0.1148	0.071*	
C28	1.0759 (3)	0.19022 (17)	−0.00322 (14)	0.0475 (6)	
H28	1.1671	0.2411	0.0168	0.057*	
C29	0.9446 (3)	0.17386 (16)	0.04690 (13)	0.0379 (5)	
C30	0.9538 (3)	0.24389 (16)	0.12859 (13)	0.0362 (5)	
C31	0.7872 (2)	0.41129 (16)	0.16111 (13)	0.0371 (5)	
C32	0.9035 (3)	0.49248 (17)	0.19818 (15)	0.0489 (6)	
H32	0.9449	0.4955	0.2538	0.059*	
C33	0.9609 (3)	0.57258 (18)	0.15078 (19)	0.0585 (7)	
H33	1.0414	0.6282	0.1761	0.070*	
C34	0.9032 (3)	0.57155 (19)	0.06981 (18)	0.0583 (7)	
H34	0.9472	0.6246	0.0403	0.070*	
C35	0.7779 (3)	0.49103 (18)	0.03032 (14)	0.0465 (6)	
C36	0.6976 (3)	0.4798 (2)	−0.05085 (16)	0.0583 (7)	
H36	0.7309	0.5287	−0.0862	0.070*	
C37	0.5730 (3)	0.3995 (2)	−0.07831 (15)	0.0603 (7)	
H37	0.5240	0.3942	−0.1327	0.072*	
C38	0.5143 (3)	0.3235 (2)	−0.02790 (14)	0.0522 (6)	
H38	0.4262	0.2700	−0.0476	0.063*	
C39	0.5908 (3)	0.33051 (17)	0.05099 (12)	0.0403 (5)	
C40	0.7222 (2)	0.41218 (16)	0.07868 (13)	0.0381 (5)	
C41	0.5557 (3)	0.27367 (17)	0.12338 (13)	0.0391 (5)	
C42	0.5437 (3)	0.37730 (16)	0.30692 (13)	0.0395 (5)	
C43	0.4711 (3)	0.43741 (19)	0.25804 (14)	0.0521 (6)	
H43	0.4999	0.4409	0.2028	0.062*	
C44	0.3572 (3)	0.4921 (2)	0.28967 (15)	0.0557 (6)	
H44	0.3107	0.5326	0.2563	0.067*	
C45	0.3133 (3)	0.48635 (19)	0.37064 (14)	0.0518 (6)	
C46	0.3809 (3)	0.42677 (19)	0.41970 (14)	0.0526 (6)	
H46	0.3495	0.4227	0.4744	0.063*	
C47	0.4956 (3)	0.37255 (18)	0.38851 (13)	0.0468 (6)	
H47	0.5412	0.3323	0.4225	0.056*	

### Atomic displacement parameters (Å^2^)

**Table:**

	*U*^11^	*U*^22^	*U*^33^	*U*^12^	*U*^13^	*U*^23^
Br1	0.0915 (3)	0.1514 (3)	0.05390 (18)	0.0842 (2)	−0.00073 (15)	−0.01430 (18)
O1	0.0969 (15)	0.1157 (16)	0.0604 (12)	0.0642 (13)	0.0364 (10)	0.0481 (11)
O2	0.0416 (9)	0.0476 (9)	0.0523 (9)	0.0028 (8)	0.0088 (7)	0.0040 (7)
O3	0.0396 (9)	0.0632 (11)	0.0616 (10)	0.0055 (8)	0.0040 (8)	0.0183 (9)
N1	0.0533 (11)	0.0609 (12)	0.0302 (9)	0.0304 (10)	0.0087 (8)	0.0161 (8)
C1	0.0566 (15)	0.0559 (14)	0.0440 (13)	0.0241 (12)	0.0071 (11)	0.0147 (11)
C2	0.0626 (16)	0.0621 (16)	0.0468 (14)	0.0263 (13)	0.0148 (12)	0.0198 (12)
C3	0.0563 (15)	0.0495 (14)	0.0478 (13)	0.0226 (12)	0.0117 (11)	0.0162 (11)
C4	0.0752 (18)	0.0665 (17)	0.0508 (14)	0.0324 (14)	0.0112 (13)	0.0183 (13)
C5	0.080 (2)	0.080 (2)	0.0722 (19)	0.0447 (16)	0.0110 (15)	0.0236 (16)
C6	0.090 (2)	0.091 (2)	0.081 (2)	0.0588 (18)	0.0273 (17)	0.0196 (17)
C7	0.096 (2)	0.096 (2)	0.0582 (17)	0.0569 (19)	0.0241 (16)	0.0151 (16)
C8	0.0745 (18)	0.0646 (16)	0.0513 (15)	0.0318 (14)	0.0119 (13)	0.0119 (13)
C9	0.104 (2)	0.114 (2)	0.0448 (15)	0.063 (2)	0.0106 (15)	0.0131 (16)
C10	0.0779 (19)	0.088 (2)	0.0482 (14)	0.0444 (16)	0.0145 (13)	0.0246 (14)
C11	0.0591 (15)	0.0617 (15)	0.0477 (13)	0.0267 (13)	0.0123 (11)	0.0216 (12)
C12	0.0500 (14)	0.0508 (14)	0.0411 (12)	0.0211 (11)	0.0058 (10)	0.0117 (11)
C13	0.0416 (13)	0.0516 (14)	0.0523 (13)	0.0169 (11)	0.0066 (10)	0.0118 (11)
C14	0.0432 (13)	0.0448 (13)	0.0451 (12)	0.0107 (10)	0.0042 (10)	0.0115 (10)
C15	0.0436 (13)	0.0444 (12)	0.0303 (10)	0.0145 (10)	0.0000 (9)	0.0067 (9)
C16	0.0442 (13)	0.0684 (16)	0.0517 (14)	0.0223 (12)	0.0117 (11)	0.0240 (12)
C17	0.0568 (15)	0.0588 (15)	0.0535 (14)	0.0218 (12)	0.0141 (12)	0.0287 (12)
C18	0.0408 (12)	0.0436 (12)	0.0334 (11)	0.0142 (10)	0.0002 (9)	0.0092 (9)
C19	0.0339 (11)	0.0373 (11)	0.0311 (10)	0.0089 (9)	0.0039 (8)	0.0086 (9)
C20	0.0387 (12)	0.0416 (12)	0.0299 (10)	0.0131 (9)	0.0048 (9)	0.0102 (9)
C21	0.0543 (14)	0.0582 (14)	0.0314 (11)	0.0267 (11)	0.0043 (10)	0.0110 (10)
C22	0.0412 (12)	0.0438 (13)	0.0432 (12)	0.0088 (10)	0.0059 (10)	0.0134 (10)
C23	0.0477 (14)	0.0438 (13)	0.0514 (14)	0.0044 (11)	−0.0003 (11)	0.0010 (11)
C24	0.0454 (13)	0.0430 (13)	0.0388 (12)	0.0139 (11)	0.0004 (10)	0.0076 (10)
C25	0.0597 (16)	0.0545 (15)	0.0502 (14)	0.0137 (12)	−0.0052 (12)	−0.0008 (12)
C26	0.082 (2)	0.0701 (18)	0.0397 (13)	0.0290 (16)	0.0080 (13)	0.0003 (12)
C27	0.0720 (18)	0.0612 (16)	0.0499 (14)	0.0205 (15)	0.0244 (13)	0.0098 (13)
C28	0.0563 (15)	0.0430 (13)	0.0490 (13)	0.0158 (11)	0.0153 (11)	0.0145 (11)
C29	0.0454 (13)	0.0377 (12)	0.0358 (11)	0.0163 (10)	0.0051 (9)	0.0111 (9)
C30	0.0388 (12)	0.0361 (12)	0.0373 (11)	0.0117 (10)	0.0023 (9)	0.0126 (9)
C31	0.0382 (12)	0.0397 (12)	0.0375 (11)	0.0152 (10)	0.0072 (9)	0.0077 (9)
C32	0.0509 (14)	0.0423 (13)	0.0537 (14)	0.0135 (11)	0.0007 (11)	0.0035 (11)
C33	0.0532 (15)	0.0380 (14)	0.084 (2)	0.0088 (11)	0.0119 (14)	0.0063 (13)
C34	0.0606 (16)	0.0446 (14)	0.0810 (19)	0.0212 (13)	0.0266 (15)	0.0267 (14)
C35	0.0511 (14)	0.0504 (14)	0.0496 (13)	0.0267 (12)	0.0180 (11)	0.0202 (11)
C36	0.0695 (18)	0.0729 (18)	0.0512 (15)	0.0384 (15)	0.0252 (13)	0.0333 (14)
C37	0.0707 (18)	0.091 (2)	0.0339 (12)	0.0396 (16)	0.0106 (12)	0.0251 (13)
C38	0.0493 (14)	0.0738 (17)	0.0377 (12)	0.0232 (12)	0.0017 (10)	0.0080 (12)
C39	0.0406 (13)	0.0536 (14)	0.0332 (11)	0.0214 (11)	0.0067 (9)	0.0111 (10)
C40	0.0399 (12)	0.0443 (12)	0.0375 (11)	0.0208 (10)	0.0123 (9)	0.0116 (10)
C41	0.0372 (12)	0.0443 (13)	0.0386 (11)	0.0127 (11)	0.0049 (9)	0.0091 (10)
C42	0.0412 (12)	0.0469 (13)	0.0338 (11)	0.0153 (10)	0.0043 (9)	0.0079 (10)
C43	0.0595 (15)	0.0697 (16)	0.0389 (12)	0.0335 (13)	0.0130 (11)	0.0174 (11)
C44	0.0607 (16)	0.0712 (17)	0.0461 (13)	0.0363 (13)	0.0054 (11)	0.0136 (12)
C45	0.0482 (14)	0.0669 (16)	0.0414 (13)	0.0256 (12)	0.0000 (10)	−0.0060 (11)
C46	0.0576 (15)	0.0696 (16)	0.0335 (12)	0.0217 (13)	0.0089 (11)	0.0035 (11)
C47	0.0525 (14)	0.0578 (14)	0.0353 (12)	0.0220 (12)	0.0038 (10)	0.0103 (10)

### Geometric parameters (Å, °)

**Table:**

Br1—C45	1.898 (2)	C21—H21A	0.9700
O1—C2	1.214 (3)	C21—H21B	0.9700
O2—C30	1.211 (2)	C22—C23	1.519 (3)
O3—C41	1.204 (2)	C22—H22A	0.9700
N1—C42	1.386 (3)	C22—H22B	0.9700
N1—C20	1.446 (2)	C23—C24	1.492 (3)
N1—C21	1.446 (3)	C23—H23A	0.9700
C1—C11	1.323 (3)	C23—H23B	0.9700
C1—C2	1.494 (3)	C24—C29	1.382 (3)
C1—C10	1.505 (3)	C24—C25	1.398 (3)
C2—C3	1.486 (3)	C25—C26	1.373 (4)
C3—C4	1.373 (3)	C25—H25	0.9300
C3—C8	1.392 (3)	C26—C27	1.365 (4)
C4—C5	1.379 (4)	C26—H26	0.9300
C4—H4	0.9300	C27—C28	1.364 (3)
C5—C6	1.368 (4)	C27—H27	0.9300
C5—H5	0.9300	C28—C29	1.396 (3)
C6—C7	1.370 (4)	C28—H28	0.9300
C6—H6	0.9300	C29—C30	1.479 (3)
C7—C8	1.387 (4)	C31—C32	1.352 (3)
C7—H7	0.9300	C31—C40	1.403 (3)
C8—C9	1.506 (4)	C32—C33	1.413 (3)
C9—C10	1.504 (4)	C32—H32	0.9300
C9—H9A	0.9700	C33—C34	1.356 (4)
C9—H9B	0.9700	C33—H33	0.9300
C10—H10A	0.9700	C34—C35	1.402 (3)
C10—H10B	0.9700	C34—H34	0.9300
C11—C12	1.468 (3)	C35—C36	1.408 (3)
C11—H11	0.9300	C35—C40	1.408 (3)
C12—C17	1.378 (3)	C36—C37	1.350 (4)
C12—C13	1.392 (3)	C36—H36	0.9300
C13—C14	1.381 (3)	C37—C38	1.408 (3)
C13—H13	0.9300	C37—H37	0.9300
C14—C15	1.381 (3)	C38—C39	1.369 (3)
C14—H14	0.9300	C38—H38	0.9300
C15—C16	1.388 (3)	C39—C40	1.397 (3)
C15—C18	1.516 (3)	C39—C41	1.477 (3)
C16—C17	1.383 (3)	C42—C47	1.389 (3)
C16—H16	0.9300	C42—C43	1.389 (3)
C17—H17	0.9300	C43—C44	1.380 (3)
C18—C21	1.509 (3)	C43—H43	0.9300
C18—C19	1.563 (3)	C44—C45	1.369 (3)
C18—H18	0.9800	C44—H44	0.9300
C19—C22	1.533 (3)	C45—C46	1.364 (3)
C19—C30	1.542 (3)	C46—C47	1.379 (3)
C19—C20	1.602 (3)	C46—H46	0.9300
C20—C31	1.518 (3)	C47—H47	0.9300
C20—C41	1.568 (3)		
			
C42—N1—C20	124.33 (16)	C23—C22—C19	116.47 (17)
C42—N1—C21	121.74 (16)	C23—C22—H22A	108.2
C20—N1—C21	113.88 (16)	C19—C22—H22A	108.2
C11—C1—C2	118.1 (2)	C23—C22—H22B	108.2
C11—C1—C10	125.0 (2)	C19—C22—H22B	108.2
C2—C1—C10	116.9 (2)	H22A—C22—H22B	107.3
O1—C2—C3	120.3 (2)	C24—C23—C22	113.39 (18)
O1—C2—C1	122.0 (2)	C24—C23—H23A	108.9
C3—C2—C1	117.63 (19)	C22—C23—H23A	108.9
C4—C3—C8	120.0 (2)	C24—C23—H23B	108.9
C4—C3—C2	119.8 (2)	C22—C23—H23B	108.9
C8—C3—C2	120.2 (2)	H23A—C23—H23B	107.7
C3—C4—C5	121.2 (2)	C29—C24—C25	118.5 (2)
C3—C4—H4	119.4	C29—C24—C23	120.92 (19)
C5—C4—H4	119.4	C25—C24—C23	120.5 (2)
C6—C5—C4	118.9 (3)	C26—C25—C24	120.4 (2)
C6—C5—H5	120.6	C26—C25—H25	119.8
C4—C5—H5	120.6	C24—C25—H25	119.8
C5—C6—C7	120.7 (3)	C27—C26—C25	120.6 (2)
C5—C6—H6	119.6	C27—C26—H26	119.7
C7—C6—H6	119.6	C25—C26—H26	119.7
C6—C7—C8	121.0 (3)	C28—C27—C26	120.0 (2)
C6—C7—H7	119.5	C28—C27—H27	120.0
C8—C7—H7	119.5	C26—C27—H27	120.0
C7—C8—C3	118.2 (2)	C27—C28—C29	120.4 (2)
C7—C8—C9	122.3 (2)	C27—C28—H28	119.8
C3—C8—C9	119.5 (2)	C29—C28—H28	119.8
C10—C9—C8	111.3 (2)	C24—C29—C28	119.9 (2)
C10—C9—H9A	109.4	C24—C29—C30	122.51 (19)
C8—C9—H9A	109.4	C28—C29—C30	117.6 (2)
C10—C9—H9B	109.4	O2—C30—C29	120.32 (19)
C8—C9—H9B	109.4	O2—C30—C19	119.93 (18)
H9A—C9—H9B	108.0	C29—C30—C19	119.75 (18)
C9—C10—C1	111.5 (2)	C32—C31—C40	118.9 (2)
C9—C10—H10A	109.3	C32—C31—C20	132.80 (19)
C1—C10—H10A	109.3	C40—C31—C20	108.34 (18)
C9—C10—H10B	109.3	C31—C32—C33	118.7 (2)
C1—C10—H10B	109.3	C31—C32—H32	120.7
H10A—C10—H10B	108.0	C33—C32—H32	120.7
C1—C11—C12	129.9 (2)	C34—C33—C32	122.6 (2)
C1—C11—H11	115.1	C34—C33—H33	118.7
C12—C11—H11	115.1	C32—C33—H33	118.7
C17—C12—C13	117.2 (2)	C33—C34—C35	120.5 (2)
C17—C12—C11	119.1 (2)	C33—C34—H34	119.8
C13—C12—C11	123.5 (2)	C35—C34—H34	119.8
C14—C13—C12	121.3 (2)	C34—C35—C36	128.4 (2)
C14—C13—H13	119.4	C34—C35—C40	116.0 (2)
C12—C13—H13	119.4	C36—C35—C40	115.6 (2)
C13—C14—C15	121.4 (2)	C37—C36—C35	121.2 (2)
C13—C14—H14	119.3	C37—C36—H36	119.4
C15—C14—H14	119.3	C35—C36—H36	119.4
C14—C15—C16	117.46 (19)	C36—C37—C38	122.6 (2)
C14—C15—C18	120.05 (19)	C36—C37—H37	118.7
C16—C15—C18	122.48 (19)	C38—C37—H37	118.7
C17—C16—C15	121.0 (2)	C39—C38—C37	118.1 (2)
C17—C16—H16	119.5	C39—C38—H38	121.0
C15—C16—H16	119.5	C37—C38—H38	121.0
C12—C17—C16	121.7 (2)	C38—C39—C40	119.4 (2)
C12—C17—H17	119.1	C38—C39—C41	133.5 (2)
C16—C17—H17	119.1	C40—C39—C41	106.76 (17)
C21—C18—C15	116.01 (17)	C39—C40—C31	113.52 (18)
C21—C18—C19	103.72 (16)	C39—C40—C35	123.1 (2)
C15—C18—C19	115.92 (17)	C31—C40—C35	123.3 (2)
C21—C18—H18	106.9	O3—C41—C39	127.9 (2)
C15—C18—H18	106.9	O3—C41—C20	124.93 (18)
C19—C18—H18	106.9	C39—C41—C20	107.03 (17)
C22—C19—C30	110.14 (16)	N1—C42—C47	119.58 (18)
C22—C19—C18	111.45 (16)	N1—C42—C43	122.77 (18)
C30—C19—C18	109.23 (16)	C47—C42—C43	117.64 (19)
C22—C19—C20	113.50 (16)	C44—C43—C42	121.4 (2)
C30—C19—C20	112.50 (15)	C44—C43—H43	119.3
C18—C19—C20	99.56 (15)	C42—C43—H43	119.3
N1—C20—C31	115.98 (17)	C45—C44—C43	119.4 (2)
N1—C20—C41	113.12 (16)	C45—C44—H44	120.3
C31—C20—C41	101.41 (16)	C43—C44—H44	120.3
N1—C20—C19	102.76 (14)	C46—C45—C44	120.4 (2)
C31—C20—C19	113.35 (16)	C46—C45—Br1	119.69 (17)
C41—C20—C19	110.53 (16)	C44—C45—Br1	119.87 (18)
N1—C21—C18	103.74 (16)	C45—C46—C47	120.3 (2)
N1—C21—H21A	111.0	C45—C46—H46	119.9
C18—C21—H21A	111.0	C47—C46—H46	119.9
N1—C21—H21B	111.0	C46—C47—C42	120.8 (2)
C18—C21—H21B	111.0	C46—C47—H47	119.6
H21A—C21—H21B	109.0	C42—C47—H47	119.6
			
C11—C1—C2—O1	−3.7 (4)	C23—C24—C25—C26	−178.9 (2)
C10—C1—C2—O1	173.9 (3)	C24—C25—C26—C27	1.6 (4)
C11—C1—C2—C3	178.5 (2)	C25—C26—C27—C28	−1.4 (4)
C10—C1—C2—C3	−3.9 (3)	C26—C27—C28—C29	−1.1 (4)
O1—C2—C3—C4	−15.5 (4)	C25—C24—C29—C28	−3.1 (3)
C1—C2—C3—C4	162.3 (2)	C23—C24—C29—C28	176.5 (2)
O1—C2—C3—C8	165.4 (3)	C25—C24—C29—C30	176.65 (19)
C1—C2—C3—C8	−16.8 (4)	C23—C24—C29—C30	−3.8 (3)
C8—C3—C4—C5	0.2 (4)	C27—C28—C29—C24	3.4 (3)
C2—C3—C4—C5	−178.9 (2)	C27—C28—C29—C30	−176.4 (2)
C3—C4—C5—C6	−1.3 (4)	C24—C29—C30—O2	−176.99 (19)
C4—C5—C6—C7	1.0 (5)	C28—C29—C30—O2	2.8 (3)
C5—C6—C7—C8	0.4 (5)	C24—C29—C30—C19	3.4 (3)
C6—C7—C8—C3	−1.6 (5)	C28—C29—C30—C19	−176.87 (17)
C6—C7—C8—C9	179.1 (3)	C22—C19—C30—O2	−159.31 (18)
C4—C3—C8—C7	1.3 (4)	C18—C19—C30—O2	−36.6 (2)
C2—C3—C8—C7	−179.6 (3)	C20—C19—C30—O2	73.0 (2)
C4—C3—C8—C9	−179.4 (3)	C22—C19—C30—C29	20.3 (2)
C2—C3—C8—C9	−0.3 (4)	C18—C19—C30—C29	143.05 (17)
C7—C8—C9—C10	−144.1 (3)	C20—C19—C30—C29	−107.37 (19)
C3—C8—C9—C10	36.7 (4)	N1—C20—C31—C32	42.7 (3)
C8—C9—C10—C1	−55.0 (4)	C41—C20—C31—C32	165.7 (2)
C11—C1—C10—C9	−143.1 (3)	C19—C20—C31—C32	−75.8 (3)
C2—C1—C10—C9	39.5 (3)	N1—C20—C31—C40	−137.41 (18)
C2—C1—C11—C12	171.6 (2)	C41—C20—C31—C40	−14.5 (2)
C10—C1—C11—C12	−5.7 (5)	C19—C20—C31—C40	104.02 (18)
C1—C11—C12—C17	151.9 (3)	C40—C31—C32—C33	−3.1 (3)
C1—C11—C12—C13	−34.1 (4)	C20—C31—C32—C33	176.7 (2)
C17—C12—C13—C14	0.0 (3)	C31—C32—C33—C34	0.4 (4)
C11—C12—C13—C14	−174.1 (2)	C32—C33—C34—C35	2.3 (4)
C12—C13—C14—C15	−0.6 (3)	C33—C34—C35—C36	176.4 (2)
C13—C14—C15—C16	0.8 (3)	C33—C34—C35—C40	−2.0 (3)
C13—C14—C15—C18	179.3 (2)	C34—C35—C36—C37	−176.8 (2)
C14—C15—C16—C17	−0.3 (3)	C40—C35—C36—C37	1.6 (3)
C18—C15—C16—C17	−178.8 (2)	C35—C36—C37—C38	0.9 (4)
C13—C12—C17—C16	0.4 (4)	C36—C37—C38—C39	−2.0 (4)
C11—C12—C17—C16	174.8 (2)	C37—C38—C39—C40	0.3 (3)
C15—C16—C17—C12	−0.3 (4)	C37—C38—C39—C41	172.9 (2)
C14—C15—C18—C21	−153.2 (2)	C38—C39—C40—C31	178.84 (19)
C16—C15—C18—C21	25.2 (3)	C41—C39—C40—C31	4.5 (2)
C14—C15—C18—C19	84.7 (2)	C38—C39—C40—C35	2.3 (3)
C16—C15—C18—C19	−96.8 (2)	C41—C39—C40—C35	−172.05 (19)
C21—C18—C19—C22	−80.1 (2)	C32—C31—C40—C39	−173.09 (19)
C15—C18—C19—C22	48.2 (2)	C20—C31—C40—C39	7.0 (2)
C21—C18—C19—C30	157.96 (17)	C32—C31—C40—C35	3.4 (3)
C15—C18—C19—C30	−73.7 (2)	C20—C31—C40—C35	−176.43 (18)
C21—C18—C19—C20	39.94 (19)	C34—C35—C40—C39	175.38 (19)
C15—C18—C19—C20	168.29 (17)	C36—C35—C40—C39	−3.2 (3)
C42—N1—C20—C31	65.1 (3)	C34—C35—C40—C31	−0.8 (3)
C21—N1—C20—C31	−112.3 (2)	C36—C35—C40—C31	−179.42 (19)
C42—N1—C20—C41	−51.5 (3)	C38—C39—C41—O3	−10.8 (4)
C21—N1—C20—C41	131.11 (19)	C40—C39—C41—O3	162.4 (2)
C42—N1—C20—C19	−170.68 (19)	C38—C39—C41—C20	173.1 (2)
C21—N1—C20—C19	11.9 (2)	C40—C39—C41—C20	−13.7 (2)
C22—C19—C20—N1	87.33 (19)	N1—C20—C41—O3	−34.4 (3)
C30—C19—C20—N1	−146.76 (17)	C31—C20—C41—O3	−159.3 (2)
C18—C19—C20—N1	−31.20 (19)	C19—C20—C41—O3	80.2 (2)
C22—C19—C20—C31	−146.72 (17)	N1—C20—C41—C39	141.88 (17)
C30—C19—C20—C31	−20.8 (2)	C31—C20—C41—C39	17.0 (2)
C18—C19—C20—C31	94.75 (18)	C19—C20—C41—C39	−103.51 (18)
C22—C19—C20—C41	−33.6 (2)	C20—N1—C42—C47	172.7 (2)
C30—C19—C20—C41	92.26 (19)	C21—N1—C42—C47	−10.1 (3)
C18—C19—C20—C41	−152.18 (16)	C20—N1—C42—C43	−6.5 (3)
C42—N1—C21—C18	−163.82 (19)	C21—N1—C42—C43	170.7 (2)
C20—N1—C21—C18	13.7 (2)	N1—C42—C43—C44	−179.6 (2)
C15—C18—C21—N1	−162.26 (18)	C47—C42—C43—C44	1.1 (4)
C19—C18—C21—N1	−34.0 (2)	C42—C43—C44—C45	−0.7 (4)
C30—C19—C22—C23	−44.8 (2)	C43—C44—C45—C46	−0.2 (4)
C18—C19—C22—C23	−166.22 (18)	C43—C44—C45—Br1	179.39 (19)
C20—C19—C22—C23	82.3 (2)	C44—C45—C46—C47	0.7 (4)
C19—C22—C23—C24	45.9 (3)	Br1—C45—C46—C47	−178.94 (18)
C22—C23—C24—C29	−20.2 (3)	C45—C46—C47—C42	−0.2 (4)
C22—C23—C24—C25	159.3 (2)	N1—C42—C47—C46	−180.0 (2)
C29—C24—C25—C26	0.7 (3)	C43—C42—C47—C46	−0.7 (3)

### Hydrogen-bond geometry (Å, °)

**Table:**

Cg1 and Cg2 are the centroids of the C3–C8 and C12–C17 rings, respectively.

**Table:**

*D*—H···*A*	*D*—H	H···*A*	*D*···*A*	*D*—H···*A*
C22—H22B···O3	0.97	2.55	3.026 (3)	111
C23—H23B···O3	0.97	2.40	3.113 (3)	130
C17—H17···Cg1^i^	0.93	2.98	3.720 (3)	137
C26—H26···Cg2^ii^	0.93	3.00	3.925 (3)	178

Symmetry codes: (i) *x*−1, *y*, *z*; (ii) −*x*+2, −*y*, −*z*.
